# Comparison of Intramuscular or Subcutaneous Injections vs. Castration in Pigs—Impacts on Behavior and Welfare

**DOI:** 10.3390/ani6090052

**Published:** 2016-08-29

**Authors:** John McGlone, Kimberly Guay, Arlene Garcia

**Affiliations:** 1 Laboratory of Animal Behavior, Physiology and Welfare, Texas Tech University, Lubbock, TX 79409, USA; agarciam@crk.umn.edu; 2 Department of Animal Science and Veterinary Technology, Tarleton State University, Stephenville, TX 76401, USA; guay@tarleton.edu; 3 Department of Agricultural and Natural Resources, University of Minnesota, Crookston, MN 56716, USA

**Keywords:** pigs, physical castration, immunocastration, immunological castration, injection

## Abstract

**Simple Summary:**

Physical castration (PC) of piglets is a painful and stressful procedure and alternatives are being sought to improve animal well-being, such as immunological castration (IC). However, IC requires injections which may also cause pain and stress during handling. In this study, piglets and finishing pigs were placed in the following treatment groups: no handling or treatment (NO), sham-handling (SHAM), intramuscular injection (IM), subcutaneous injection (SQ), or PC on piglets only. Behavior was monitored for 1 h prior and 1 h post treatment in each age group. Social behavior and feeding behavior, and signs of pain were recorded. Physical castration caused measurable pain-like behaviors and general behavioral dysregulation at a much higher level than changes associated with handling associated with IM or SQ injections. Overall, injections did not cause a change in weaning pig behaviors. Finishing pigs given SQ injections showed a lower number of feeding behaviors post treatment but other changes were not observed in the other treatment groups.

**Abstract:**

Physical castration (PC) is painful and stressful for nursing piglets. One alternative to PC is immunological castration (IC), but the pain and stress of handling associated with injections have not been assessed. The objectives of this study were to measure the pain and distress of subcutaneous (SQ) and intramuscular (IM) injections compared to PC in piglets, and to compare SQ or IM injections in finishing pigs. After farrowing, 3 to 5 d old male piglets were randomly assigned to (control) no handling treatment (NO), sham-handling (SHAM), IM, SQ, or PC. Finishing pigs were assigned to NO, SHAM, IM, or SQ. Behavior was monitored for 1 h prior and 1 h post treatment in each age group. Social, feeding behaviors, and signs of pain were recorded. Finishing pigs treated with SQ injections had higher feeding behaviors pre-treatment than they did post-treatment. Overall, physical castrations caused measurable pain-like behaviors and general behavioral dysregulation at a much higher level than the other treatment groups. SQ and IM injections did not cause either significant behavioral or physiological alterations in piglets. SQ injections caused a decrease in finishing pig feed behaviors post treatment (*p* = 0.02) and SHAM treated finishing pigs spent significantly more time lying than the other treatment groups. In general IM and SQ injections did not cause any other significant changes in behavior or physiology.

## 1. Introduction

Physical castration (PC) of male piglets early in life is a common management practice on commercial swine farms in the U.S. PC is performed primarily to reduce the accumulation of boar taint compounds, aggressive behavior in post-pubertal male pigs, and undesirable pregnancy in co-housed females at slaughter; however, consumer attitude toward PC is becoming increasingly negative due to the known pain and distress caused by the procedure [1,2,3,4]. Attempts to reduce this discomfort by use of local or general anesthetics have been insufficient to date [1,2,3] and effective drugs have not been approved for the use in pigs in the U.S. One alternative to PC is the immunological castration (IC) of male pigs [5]. IC yields a carcass without boar taint and may improve pig welfare by reducing the stress of PC [5,6,7]; however, the stress of the IC process has not been assessed. Aside from IC, many pigs receive one or more injections during their lifetime for health reasons such as vaccinations. Increased levels of serum cortisol have been documented as a response to “unpleasant” handling experiences [8]. Robert et al. [9] reported an increase of serum cortisol concentrations in gilts and lactating sows 10 and 20 min after a subcutaneous injection of saline. One effect of a chronic elevation of plasma corticosteroids may be less favorable nitrogen imbalance, which may result in depressed growth rate [10,11]. Our longer-term goal was to define a welfare matrix for alternatives to pig physical castration procedures. Our specific goal was to determine the pain and distress in both piglets and finishing pigs receiving an injection using physiological and behavioral methods. Piglets were used to define the experience of PC compared with the stress of injection in littermates. The PC of older male pigs was not performed because it causes an unacceptably large stress response [4]. Thus, PC could not be compared directly with SQ or IM injections in older pigs. In finishing pigs (when IC would take place), a sham or saline injection was used as a control to compare SQ or IM injections.

## 2. Materials and Methods

### 2.1. Experimental Section

This experiment was conducted at the Texas Tech University (TTU) Swine Unit, New Deal, TX, USA. Commercial, high-performance lines of modern genetics (PIC, Inc., Hendersonville, TN, USA) were used in this study. This work was reviewed and approved by the Texas Tech University Animal Care and Use Committee (IACUC) before the project began (12069-09). All work was performed by IACUC trained TTU personnel. All animals were fed a diet to meet or exceed NRC nutrient requirements. Feed and water were provided *ad libitum*.

Piglets and sows were housed in farrowing crates in adjacent rooms. Both piglets and finishing pigs (barrows and gilts) used for the study were weaned at approximately 3 wks of age. After weaning, pigs were transferred to a nursery for approximately 7 wks. Piglets were kept in groups of 10 in pens (1.5 m × 2.1 m) with woven wire flooring, with one metal feeder (filled on a weekly basis) that allowed six piglets to eat simultaneously, and a wall mounted water nipple. The pigs were then moved to pens in the test finishing barn located at the same facility. Finishing pigs were kept in groups of 5 in pens (2.1 m × 3.6 m) that had fully slatted concrete floors, and metal bars dividing them from the adjacent pens. Each pen had automatic feeders that allowed for two pigs to eat simultaneously, and one wall mounted water nipple. The barn was also equipped with a mechanical ventilation system controlled by a thermostat. The nursery was kept between 26.6 °C to 32.2 °C (with the temperature being brought down as the piglets got older) and the finishing barn was kept between 21.1 °C to 22.2 °C. All pigs were kept in their thermal neutral zone.

### 2.2. Study 1: Piglet Phase

At 3 d and 5 d of age, piglets from 10 different litters were randomly assigned to treatment groups and treatments applied. Five male piglets within each litter (*n* = 50) were randomly assigned to one of the 5 treatment groups: control- no handling or treatment (NO), 1 mL saline injected intramuscularly (IM) in the neck region, 1 mL saline injected subcutaneously (SQ) in the neck region, sham-handled to mimic handling and a touch to the neck with one finger but without an injection (SHAM), and physical castration (PC) without pain relief.

Piglets in the PC group were physically castrated by 3–5 d of age by the same person by placing them upside down between the handler’s legs, exposing the anogenital region, each testicle was then pushed dorsally towards the surface of the scrotum and an incision with a sharp scalpel was made above the testicle and the testicle was then externalized. Each testicle was then grasped and pulled out, tearing the spermatic cord. Once the treatments were applied, piglets were returned to their home pen.

To prevent an impact on piglet behaviors due to handling for blood collection, blood was collected at a single time 60 min post treatment. There was one person that was assigned to bleeding the pigs. Piglets were brought over to the bleeder from each pen, making sure not to mix pigs from other pens. Piglets were taken from their home pen and placed on their backs in a V-trough with their forelegs and hind legs manually restrained by trained personnel for blood collection. It took no longer than 2 min to bleed each pig. A total of 7–10 mL of blood was collected via jugular venipuncture into vacutainers (BD Vacutainers^®^, Becton, Dickinson and Company, Franklin Lakes, NJ, USA) containing 5.4 mg of potassium EDTA. Blood samples were centrifuged and plasma was collected and frozen until further analysis for cortisol concentrations. After blood samples were collected from the piglets they were placed back into their respective farrowing crates, where piglet behavior was further observed and recorded. The procedures performed were all conducted by three personnel and they conducted the same task throughout the piglet phase of the study.

Piglet behavior was recorded and data were collected via scan sample every 15 min for each pig for all treatment groups using digital Sony^®^ camcorders DCR-SR85 (Sony, San Diego, CA, USA). The video cameras were placed on the rafts of the buildings pointing towards each pen. The video recordings were then downloaded and behavior was sampled in 15 min intervals (observers were blind to the treatment groups), in 1 h before and 1 h post periods. Trained observers were validated, the correlation between observers was over 90% and means were not different between one observer and the other.

Behaviors recorded at 3 d to 5 d of age included lying with sow contact, lying without sow contact, sitting, standing, walking, nursing, and signs of pain (standing hunched over, shivering). All behaviors were described in detail and defined according to a previous report [12].

### 2.3. Study 2: Finishing Pig Phase

Finishing pigs were approximately 20 weeks of age and were randomly chosen from 10 random pens within the finishing barn. Four male pigs within each pen (*n* = 40) were assigned to one of four treatment groups: control- no handling or treatment (NO); sham-handled to mimic handling and a touch to the neck with one finger but without an injection (SHAM), 2 mL saline injected intramuscularly (IM), and 2 mL saline injected subcutaneously (SQ). Pigs were not removed from their home pens. A sorting board was used to isolate each pig and give it the treatment. The procedures were performed by three personnel, one cornered the pigs with the sorting board, one snared the pigs, and the other applied the respective treatments.

To prevent an impact on pig behaviors due to handling for blood collection, blood was collected 60 min post treatment. There was one person that was assigned to bleeding the pigs. Blood samples were taken from each pigs in their pens. Pigs were restrained with a snare and a total of 10 mL of blood was collected via jugular venipuncture into vacutainers (BD Vacutainers^®^, Becton, Dickinson and Company, Franklin Lakes, NJ, USA) containing 5.4 mg of K2 EDTA. It took no longer than 2 min to bleed each pig. Blood samples were centrifuged and plasma was collected and frozen until further analysis for cortisol concentrations.

Pigs were videotaped and behavior was sampled (observers were blind to the treatment groups) in 15 min intervals (observers were blind to treatment groups), in 1 h before and 1 h post periods (all four treatments were administered concurrently). Behaviors recorded included lying, eating, sitting, standing, walking, and drinking.

### 2.4. Hormonal Assay

Serum cortisol concentrations were measured using commercial radio-immunoassay kits (Enzo Life Sciences, Farmingdale, NY, USA). All samples were analyzed in duplicate. The sensitivity level of the assay system was 57 pg/mL. The inter-assay coefficient of variation was 10.8%. The intra-assay coefficient of variation ranged from 8.5% to 9.3%.

### 2.5. Statistical Analyses

For both studies, data were analyzed using the GLM procedure of SAS (SAS Inst., Inc., Cary, NC, USA). The experimental design was a randomized complete block design, with a split-plot over time (time being 15 min intervals, 1 h before and 1 h after treatment for a total of eight periods) and five males within a litter served as the experimental unit. The residuals were tested for normal distribution using the univariate procedure of SAS. The statistical model included the main fixed effects, treatment, period, litter, and all possible interactions. Litters or pens were used as blocks (each treatment was found in each block). The treatment by block term was used as the error term to test treatment effects. Data for piglets and finishing pigs were analyzed separately. A predicted difference test was used for multiple comparisons using the PDIFF option in SAS.

## 3. Results

### 3.1. Study 1: Piglet Phase

In the hour prior to treatment, piglets did not show differences in behavior among treatments. During the hour after treatment, the highest percent of behaviors displayed by the treatment groups were for lying in contact with the sow (Table 1).

**Figure 1:**
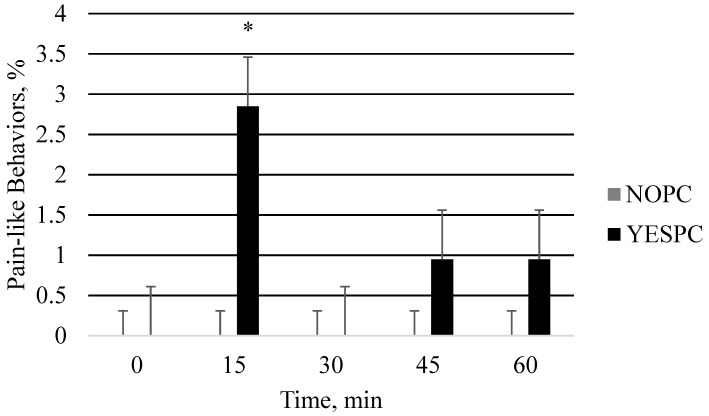
Least squares means for pain-like behaviors in piglets (*p* = 0.07): not physically castrated (NOPC) and physically castrated (YESPC). *N* = 50 piglet observations. * Between treatments, indicates a significant difference in means (*p* < 0.05).

Physically castrated pigs tended to exhibit more pain-like behaviors and general behavioral dysregulation than the other treatment groups (Figure 1). Pain-like behaviors in PC pigs were higher during the 15 min period after castration compared to all other time periods (*p* = 0.01). Pain-like behaviors remained similar from 45 min to 60 min. No injection site skin reactions were observed with IM or SQ injections.

### 3.2. Study 2: Finishing Pig Phase

Finishing pigs exhibited little to no changes in behavior post-treatment (Table 2). The only changes seen were for lying and eating behaviors. SHAM finishing pigs spent more time lying than NO (*p* = 0.001), IM (*p* = 0.02), and SQ pigs (*p* = 0.03). However, lying behaviors among NO, IM, and SQ groups were not different (*p* > 0.05). Additionally, no injection site skin reactions were observed with IM or SQ injections.

**Figure 2:**
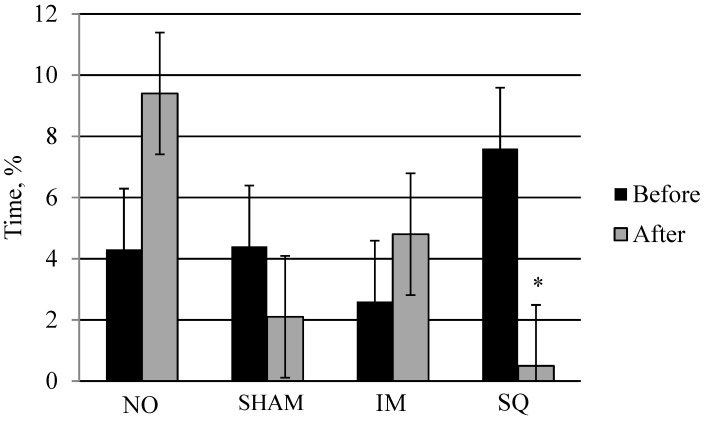
Least squares means for percent of time finishing pigs spent eating before and after receiving treatment (*p* < 0.05): none (NO), Sham (SHAM), intramuscular (IM) injection, or subcutaneous (SQ) injection. *N* = 40 pig observations. * Within treatment, indicates a significant difference in means (*p* < 0.05).

Drinking behaviors were not different among treatment groups (*p* = 0.62). There was a treatment by period effect for feeding (*p* = 0.03; Figure 2). Feeding behavior in the before period was higher than in the after period for SQ pigs (*p* = 0.02). NO pigs tended to eat less in the before period than in the after period. SHAM and IM pigs showed no differences in eating behaviors in the before and after periods. When comparing pigs across treatments in the before period, SQ pigs tended to eat more than IM pigs, but there were no differences between the SQ pigs and the other treatment groups. When comparing treatment groups in the after period, NO pigs had a higher percentage of feeding behaviors compared to SHAM (*p* = 0.02) and SQ pigs (*p* = 0.001), but were not different than IM pigs (*p* = 0.12). Furthermore, cortisol levels were not different among treatment groups (*p* > 0.05; Table 2).

## 4. Discussion

Behavioral changes in piglets and finishing pigs in stressful situations are common. Changes in behaviors such as lying, standing, sitting, eating/suckling, drinking, and vocalizing have been reported to change during/after castration [4,13], loading and unloading ramps [14], transport [14,15,16,17], after processing [18], and under other circumstances. Novelty and handling itself can be stressful and lead to physiological [19] and behavioral changes [1,4,20,21].

Handling and injections have been reported to cause acute stress [8,9], adding that cortisol levels returned to normal within 24 h of an initial injection or handling bout. Hemsworth et al. [22] found that injection treatments in grower pigs over a period of 3 weeks caused some changes in behavior and moderate increases in cortisol but were not different than the results of control pigs. They concluded that there is neither physiological nor production evidence to indicate injection treatments affect long term stress physiology. Literature pertaining to pain related responses in piglets due to different types of injections (IM, SQ, IV) is limited. Pain-related responses due to routine husbandry procedures have been widely reported [18,23,24,25]. Thus, the current findings can be compared to the literature referring to pain related responses in husbandry procedures.

In contrast to our findings in piglets, where lying behaviors were not different among treatment groups, SHAM processed piglets (ear notched and tail docked) have been reported to spend less time lying and more time standing than control pigs [18]. Castrated pigs have been reported to vocalize at a higher frequency than SHAM castrated pigs [26,27]. Castration has been shown to be painful regardless of the age in which it is done [28]. In the current study, vocalizations were recorded post-treatment, however, vocalizations were not significantly different among treatment. Research has indicated that pain-related call types (grunts, squeals, screams) in piglets can be identified and used as evidence for pain related use of different call types [24]. Therefore, the use of piglet call types may also be used to measure discomfort of different procedures in piglets. In future studies, it may be beneficial to include vocalizations of pigs during procedures that cause acute pain, such as injections.

Studies conducted on the use of local anesthetics administered into the testicles of pigs have suggested that these injections produce as much or more pain/stress than the surgery itself [28,29]. Therefore, the type of injection and the injection site location may lead to pain. However, since the number of studies relating to pain of injections is limited and mostly compare the increase in welfare of immunological castration (GnRF-conjugate injections) to physical castration it is important to turn some attention to the effects of solely injecting animals and the effects or consequences they may have. Considering the fact that during the lifetime of a slaughter pig, it has to be injected 2 or 3 times, injection of heavy pigs can be very stressful for the pigs, but also for the farmer, which may lead “failures” due to ineffective injections.

Although, we failed to see any reaction at the injection site, immunological castration may cause a skin reaction and tissue damage away from the injection site [30], along with the possibility of abscesses being produced at the site of injection. Other negative consequences may develop if the same needles are used continuously without changing them (which may be commonly done at some facilities with automatic injectors).

In the current study, PC piglets tended to display more pain-related responses than the other treatment groups. Pain-like behaviors in PC piglets were significantly higher in the 15 min period after castration, but began to decrease and remained similar from the 45–60 min periods after castration. In the finishing pigs, there were no differences in pain-related responses among treatment groups. If cortisol is high to begin with as may be the case in animals that are not commonly handled then it is difficult to identify the increase in cortisol concentrations due to pain. This is commonly seen in studies during transportation of pigs [14,31,32]. Thereby, in the current study cortisol concentrations may have been masked due to handling because basal cortisol levels were increased (40–75 ng/mL). However, injection did not cause a rise in cortisol above the level caused by handling. Possible ways to diminish this handling effect in the future may be to have a catheter in place for blood drawing. Nonetheless, the stress of physical castration is well known to affect behavior of piglets [1,4,20,27] due to the pain related to castration, which can last for several hours to days [13]. Additionally, post-treatment behaviors in the current study may not have been recorded long enough to see a significant effect of castration on piglets.

Pain-related responses due to injections among treatment groups were not observed. It was expected that injections would cause some pain and discomfort and that the pigs would be reluctant to move thus, decreasing standing, walking, drinking and eating behaviors and increasing lying behaviors. It may have been that the pigs were not observed long enough to see tenderness/pain associated with injections, since they were observed for only one hour post treatment. In addition, basal cortisol levels were elevated indicating that handling associated with blood collection may have masked cortisol differences among treatment groups. Garcia et al. [14,32] reported that handling and blood sampling via jugular venipuncture every 8 h for 32 h may have masked the stress weaned pigs experienced during 32 h of transport, as cortisol levels were similar among treatment groups being both transported and non-transported with or without feed and water. Cortisol data in this study showed no differences in the concentrations between injections and physical castration. Since the first report that PC of piglets caused pain-induced behavioral changes, the painful effects of physical castration have been replicated [1,33]. Pharmacological methods to reduce pain are further complicated by the approval process required by the FDA, or other governmental entities, before these analgesics can be used in food animals.

Behavioral responses in finishing pigs were minimal. Lying behaviors in finishing pigs were higher in SHAM pigs than in NO, IM, and SQ pigs. This may suggest that handling alone may have caused some behavioral effects in some of the pigs that were difficult to handle. Inactivity after handling is sometimes common in finishing pigs, especially when they are excessively handled. Handling in addition to treatment could cause higher numbers of lying behaviors in treatments, especially in those animals that received injections, due to possible tenderness to the area causing a reluctance to move. Although we only observed behaviors for 1 h post-treatment, the current findings are in agreement to previous findings who reported that SQ injections of Improvac^®^ caused an increase in inactivity in non-physically castrated pigs during the days after the first injection [34]. This may have been either due to the pain caused by inflammatory reactions to the SQ injection or early effects of the immunization on the endocrine system. IC can cause an increase in feeding behaviors and a reduction in general activity [35].

IM pigs seemed to have similar behaviors to NO pigs, as seen in their overall eating behaviors post-treatment, but most behaviors exhibited by IM pigs were not different than SQ and SHAM pigs.

Feeding behaviors in the before period were significantly higher than the after period in SQ pigs. It may have been coincidence in that SQ pigs may have eaten more in the before period and were possibly full in the after period, thus eating less. Additionally, pigs spent a similar amount of time feeding, but at different time points. These findings may also suggest that SQ pigs may have experienced some pain or discomfort, and therefore, a small period of inappetence due to the injection was observed. In 10 year old children, less redness, swelling, itching, and pain have been seen with IM injections compared to SQ injections [36]. Regardless, injections and handling caused little to no alteration in behavior in the post period of the current study.

In humans, injections with a low pH solution can produce pain and hyperalgesia, mechanical hyperalgesia in in vivo rats, and increased firing of nociceptors in vitro using isolated nerve-skin preparations [37]. For future studies, it may be beneficial to consider the pH of injections and possibly observe behavioral changes over longer periods of time after injections, as this study only observed behavior for 1 h post-treatment.

## 5. Conclusions

SQ and IM injections did not cause any measurable changes in behavior nor physiology of piglets. Injections given SQ caused a decrease in finishing pig feeding behaviors post treatment but no other alterations in behavior nor physiology were observed. Pain assessment in animals is difficult and the use of behavioral and psychological scores can quantify the severity of pain and distress [38]. Although, behavior in piglets was not adversely affected according to these findings, physically castrated piglets tended to show more pain-like behaviors than the other treatment groups. Pain caused by physical castration has been widely documented, however, handling alone has also been seen to cause stress in pigs, which may be the reason that behavior and cortisol measures were similar throughout the treatment groups. Further studies looking at the effects of injections possibly involving call types to measure pain and acute stress, pH of injections, and extended behavioral observations should be considered and would be a benefit to the industry. Considering that the pig industry is being faced with high animal welfare standards and that physical castration may be subjugated by immunological castration in the near future, the industry would highly benefit from knowing how injections physiologically and behaviorally affect pigs. We conclude that only a more thorough investigation, including aspects of behavior and physiology of pigs both physically castrated and immunologically castrated could more clearly define if welfare can be improved.

## Figures and Tables

**Table 1:** Least squares means for percent of time piglets spent exhibiting behaviors for combined before and after treatment periods and cortisol concentrations for the 1 h after treatment period. Periods (PER) were divided into 1 h before treatment and 1 h after treatment. Treatments (TRT) applied at 3 to 5 d of age: no handling or treatment (NO), intramuscular (IM) injection, subcutaneous (SQ) injection, nothing, SHAM handling or physically castrated (PC). *N* = 50 piglet observations.

Behavior	NO	SHAM	IM	SQ	PC	SE	*p*-Value
Lying with contact	60.4	60.8	61.2	57.4	53.9	2.17	0.15
Lying without contact	5.0	3.2	2.0	1.9	8.8 ^a^	1.80	0.09
Sitting	1.4	2.5	2.4	2.9	2.1	0.69	0.57
Standing	7.9	8.3	11.0	9.4	10.2	1.46	0.72
Walking	8.5	7.5	7.6	6.7	7.8	1.80	0.88
Nursing/Eating	16.9	17.6	15.7	21.8	16.4	2.39	0.46
Exhibiting pain	0.0	0.0	0.0	0.0	0.60 ^b^	0.18	0.07
Cortisol (ng/mL)	75.8	43.5	55.5	53.7	46.1	12.3	0.34

^a^ Overall, the treatment effect was a trend, however this Least Squares mean differs from the other treatments, *p* < 0.05, by the predicted difference test. ^b^ See Figure 1 for these data over time.

**Table 2:** Least squares means for percent of time finishing pigs spent exhibiting behaviors for combined before and after treatment periods and cortisol concentrations for 1 h after treatment period. Periods (PER) were divided into 1 h before treatment and 1 h after treatment. Treatments (TRT): touch to neck (SHAM), intramuscular (IM) injection, subcutaneous (SQ) injection, or nothing (NO). *N* = 40 pig observations.

Behavior	NO	SHAM	IM	SQ	SE	*p*-Value TRT	*p*-Value TRT × PER
Lying	77.7 ^a^	87.6 ^b^	80.0 ^a^	80.7 ^a^	2.26	0.05	0.68
Eating	6.9	3.1	3.7	4.1	1.69	0.38	0.03 *
Sitting	0.55	0.13	0.96	1.6	0.49	0.21	0.71
Standing	6.0	3.3	4.5	6.1	1.13	0.14	0.81
Walking	6.6	3.9	7.0	5.6	1.31	0.30	0.56
Drinking	1.9	0.7	3.2	2.6	0.6	0.11	0.62
Cortisol, ng/mL	5.4	10.5	5.1	13.1	3.10	0.19	--

^a,b^ Tends to differ from other treatments, *p* < 0.05. * Eating behaviors within treatment groups were different over time, *p* < 0.05. See Figure 2 for TRT*PER interaction for eating behaviors. -- No *p*-value is available for TRT*PER because cortisol was only measured after pigs received treatment.
